# Excess Secretion of Gel-Forming Mucins and Associated Innate Defense Proteins with Defective Mucin Un-Packaging Underpin Gallbladder Mucocele Formation in Dogs

**DOI:** 10.1371/journal.pone.0138988

**Published:** 2015-09-28

**Authors:** Mehmet Kesimer, John Cullen, Rui Cao, Giorgia Radicioni, Kyle G. Mathews, Gabriela Seiler, Jody L. Gookin

**Affiliations:** 1 Department of Pathology and Laboratory Medicine and Cystic Fibrosis/Pulmonary Research and Treatment Center, University of North Carolina at Chapel Hill, Chapel Hill, North Carolina, United States of America; 2 Department of Population Health and Pathobiology, College of Veterinary Medicine, North Carolina State University, Raleigh, North Carolina, United States of America; 3 Department of Clinical Sciences, College of Veterinary Medicine, Center for Comparative Medicine and Translational Research, North Carolina State University, Raleigh, North Carolina, United States of America; 4 Department of Molecular Biomedical Sciences, College of Veterinary Medicine, North Carolina State University, Raleigh, North Carolina, United States of America; University of California, Merced, UNITED STATES

## Abstract

Mucosal protection of the gallbladder is vital yet we know very little about the mechanisms involved. In domestic dogs, an emergent syndrome referred to as gallbladder mucocele formation is characterized by excessive secretion of abnormal mucus that results in obstruction and rupture of the gallbladder. The cause of gallbladder mucocele formation is unknown. In these first mechanistic studies of this disease, we investigated normal and mucocele-forming dog gallbladders to determine the source, identity, biophysical properties, and protein associates of the culprit mucins with aim to identify causes for abnormal mucus behavior. We established that mucocele formation involves an adoptive excess secretion of gel forming mucins with abnormal properties by the gallbladder epithelium. The mucus is characterized by a disproportionally significant increase in Muc5ac relative to Muc5b, defective mucin un-packaging, and mucin-interacting innate defense proteins that are capable of dramatically altering the physical and functional properties of mucus. These findings provide an explanation for abnormal mucus behavior and based on similarity to mucus observed in the airways of people with cystic fibrosis, suggest that abnormal mechanisms for maintenance of gallbladder epithelial hydration may be an instigating factor for mucocele formation in dogs.

## Introduction

The gallbladder is lined by a layer of epithelial cells that serve at the frontline of defense against bile; one of the most noxious productions by the human body. Bile is produced by the liver and is the major excretory route for lipophilic xenobiotics and endogenous waste products and serves as a carrier for delivery of bile acids needed for dietary fat assimilation. In addition to providing a physical barrier for containment of bile, the gallbladder epithelium plays a key role in transport of water and electrolytes, acidification of bile, and reabsorption of cholesterol and other bile lipids. The integrity of the epithelium and its functions are protected by secretion of mucins that serve as a barrier against exposure to lumen bile solutes and bile acids. Mucus contains hundreds of structural and protective proteins and glycoproteins including highly oligomeric mucin macromolecules that provide an infrastructure to the mucosal surface and influence the rheological properties of the mucus gel.

There are 4 major gel-forming mucins found at human mucosal surfaces, MUC2, MUC5AC, MUC5B, and MUC6. Their localization in the body depends on the functional requirements of the epithelial barrier. For instance MUC5B is characteristic of transportable mucus and predominates on respiratory mucosa, while MUC5AC and MUC2 form a firm mucus and predominate in hostile environments such as the gastric and colonic mucosa [1]. Mucins are synthesized, stored and secreted from mucous cells of either the sub-mucosal glands or the surface epithelia (goblet cells)[2]. Mucins are produced in low levels in health but they are over produced in a number of hypersecretory disorders in which they can directly contribute to the pathogenesis and prognosis of disease.

Diseases of the gallbladder are the second leading cause for gastrointestinal-related hospitalizations in the United States[3]. Greater than 228,000 biliary endoscopies and 700,000 cholecystectomies are performed each year resulting in medical expenses in excess of $6.5 billion dollars[3,4]. Most of these gallbladder diseases incriminate an instigating or reactionary dysfunction of the gallbladder epithelium. In particular, abnormalities related to abnormal mucin secretion or mucus behavior are thought to contribute to the pathogenesis of gallbladder stone formation, cholecystitis, biliary cancer, and cystic fibrosis-associated gallbladder disease[5–8].

Compared to the intestinal epithelium, much less is understood regarding function of the gallbladder epithelium. In these studies we investigate a unique and emergent disease syndrome of dogs characterized by an insidious accumulation of thick, immobile, adhesive, and rubbery mucus within the gallbladder. Commonly referred to as a gallbladder mucocele, the syndrome was rarely diagnosed prior to 10 years ago and has emerged internationally as one of the most common causes of gallbladder disease in the dog[9–14]. The disease afflicts older aged dogs of many different breeds but with apparent predilection for Shetland sheepdogs[11,15], Cocker spaniels[15], Pomeranians[15], Miniature Schnauzers[15], and Chihuahuas[15]. A gallbladder mucocele is typically diagnosed in dogs at the time of abdominal ultrasonography to investigate clinical signs of gastrointestinal illness that are usually secondary to gallbladder pain, gallbladder rupture, or common bile duct obstruction caused by mucus accumulation. Although surgical removal of the gallbladder carries a good long term prognosis for survival, perioperative mortality for these dogs ranges from 7 to 45%[9–12,14].

Several predisposing factors for gallbladder mucocele formation in dogs have been identified or are suspected such as concurrent endocrinopathies[13], hyperlipidemia[11,15], and poor gallbladder motility[16]. However, the underlying cause of gallbladder mucocele formation is essentially unknown. As a basis for understanding the pathogenesis of mucocele formation in dogs, these studies are the first to investigate normal and affected gallbladders for ostensibly mechanistic causes for abnormal mucus formation. In view of this objective, here we sought to determine the source of mucin secretion, identity and properties of the mucins involved, and composition of the mucin-associated proteome participating in formation of the adhesive, rubber-like mucus that accumulates during gallbladder mucocele formation.

## Materials and Methods

### Dogs

All dogs from which a gallbladder mucocele was obtained for this study were presented by their owners to the Veterinary Hospital at North Carolina State University College of Veterinary Medicine for further diagnostic evaluation of clinical illness. Abdominal ultrasonographic examination of each dog revealed the presence of an enlarged gallbladder containing non-gravity dependent, immobile bile having a stellate or finely striated bile pattern considered pathognomonic for gallbladder mucocele formation. A definitive diagnosis of gallbladder mucocele in each dog was made based on gross and light microscopic findings of the presence of a large viscous accumulation of mucus that filled and distended the gallbladder lumen with formation by the gallbladder mucosa of long, thin and branching fronds of well-differentiated gallbladder epithelial cells containing modestly distended clear cytoplasm filled with mucin and supported by a scant amount of submucosa that extends into the mucus. Dogs from which normal gallbladders were obtained were research animals undergoing euthanasia by means of intravenous administration of pentobarbital for the purpose of colony depopulation. All animal use was carried out in accordance with the recommendations in the Guide for the Care and Use of Laboratory Animals of the United States National Institutes of Health. The protocol was approved by the Institutional Animal Care and Use Committee of North Carolina State University. The use of tissues obtained from client-owned dogs with gallbladder mucocele was permitted by each owner by means of signed informed consent.

### Gallbladder sample acquisition

Mucocele gallbladders were obtained immediately following surgical cholecystectomy in each dog. Normal gallbladders were obtained from research dogs immediately following euthanasia. The gallbladder was opened longitudinally and samples of mucoid content were snap frozen in liquid nitrogen and stored at -80°C. Full thickness sections of gallbladder wall and content were fixed in 10% neutral buffered formalin at room temperature, Trump’s formaldehyde-glutaraldehyde (4:1) at 4°C, or frozen-embedded in optimal cutting temperature media and stored at -80°C.

### Histopathology, cytochemistry, and immunohistochemistry

Full-thickness sections of gallbladder mucosa were fixed in 10% neutral buffered formalin, paraffin-embedded, and sectioned at a thickness of 7 μm prior to mounting on glass slides. Specimens were stained using Mayer-Harris hematoxylin and alcoholic eosin Y (H&E), periodic acid Schiff (PAS), alcian blue (at pH 1 and pH 2.5), and toluidine blue using a Leica Autostainer XL (Nussloch, Germany D-69226). High iron diamine (HID) and alcian blue-pyronine Y (ABPY) staining were performed as per published protocols. Hyaluronidase pre-digestion was performed using bovine (0.5 mg/ml; H-3884, Sigma) and bacterial hyaluronidase (50 IU/ml; Cat# 151270, MP Biomedicals) in acetic acid-sodium acetate buffer (2:1 v/v) at 37°C for 1 hour.

For immunohistochemistry, sections were deparaffinized in xylene, rehydrated to water using a dilution series of alcohol, and quenched with 3% hydrogen peroxide. Antigen retrieval was performed by immersion in pH 6 citrate buffer in a Pascal pressurized heating chamber according to manufacturer settings (Dako Denmark) followed by treatment with blocking serum matching the species of origin of secondary antibody. Primary antibodies used for antigen detection were monoclonal mouse anti-rat PCNA antibodies (1:3500; abcam ab29). Incubations with primary antibody were performed at room temperature for 30 minutes or 4°C overnight in a humidified chamber. Visualization of PCNA antibody was performed using a polymer detection system (Dako EnVision) and Dako Autostainer (Dako, Carpinteria CA). Diaminobenzidine was used as the chromogenic peroxidase substrate and sections were counterstained with Mayer’s or Gill Hematoxylin. Light micrographs were digitally captured using a Zeiss AxioImager M-1.

### Scanning and transmission electron microscopy

For scanning electron microscopy, samples were rinsed twice for 15 min each with 1.0 M Sorenson’s phosphate buffer (pH 7.2–7.4) and dehydrated in an ascending series of ethanol (50%, 75%, 95%, and 95%) for 15 min each, culminating in two washes in 100% ethanol for 30 min each. Samples were then dried in a Ladd critical-point dryer. Inserts were cut away and mounted with carbon tape on aluminum specimen stubs, and sputter coated with ∼20 nm of gold–palladium using an Anatch Hummer VI sputter coater. Samples were viewed using a JEOL 6360 LV scanning electron microscope. For transmission electron microscopy, samples were rinsed in 0.1 M sodium phosphate buffer (pH 7.2) and placed in 1% osmium tetroxide in the same buffer for 1 hour at room temperature. Samples were rinsed two times in distilled water and were dehydrated in an ethanol series culminating with two changes of 100% acetone. Tissues were then placed in a mixture of Spurr resin and acetone (1:1) for 30 minutes, followed by 2 hours in 100% resin with two changes. Finally, tissues were placed in fresh 100% resin in molds and were polymerized at 70°C for 8 hours to 3 days. Semithin (0.25–0.5 mm) sections were cut with glass knives and stained with 1% toluidine blue-O in 1% sodium borate. Ultrathin (70–90 nm) sections were cut with a diamond knife, stained with methanolic uranyl acetate followed by lead citrate, and examined using a FEI/Philips EM 208S transmission electron microscope.

### Agarose gel electrophoresis

Mucus samples from normal and mucocele gallbladders were solubilized in 6 M urea reduction buffer (containing 0.1 M Tris, 5 mM EDTA at pH 8.0) and treated with 10 mM DTT for 2h at 37°C. Iodoacetemide was added to a final concentration of 25 mM and the mixture was left in the dark for 30 min at room temperature. Agarose gel electrophoresis was performed in 0.7% (w/v) agarose gels. Proteins were transferred to nitrocellulose membranes prior to detection of mucins by immunoblotting. Muc5b222[17] antibody, raised against mouse Muc5b were used to detect Muc5b mucin. MUC5ACIII antibody[18] was used to probe Muc5ac mucin. Immuno-detection was performed using an infrared imaging system (Li-Cor Odyssey, Lincoln NE).

### Rate-zonal centrifugation of mucins

The maturation state of mucins was determined by rate–zonal centrifugation[19]. Mucus samples were solubilized in 4M GuHCl and layered onto a 6–8 M GuHCl gradient. Samples were spun at 40,000 rpm for 2.5 hours at 15°C in an SW40 rotor. After centrifugation fractions were collected from the top of the tubes into 12 fractions. Immunoblotting was performed after fractions were transferred to nitrocellulose membranes using slot blotting and Muc5b222 antibody.

### Gel filtration chromatography of mucus samples

Approximately 40 mg of mucus from normal and mucocele gallbladders were extracted/solubilized in 1 ml of 6M GuHCl reduction buffer (containing 100 mM Tris, 5 mM EDTA, at pH 8.0) overnight at 4°C. Samples were centrifuged for 10 min at 3000 × g and the supernatant was subjected to gel filtration chromatography on a sepharose CL2B (2.5 × 10 cm) to separate mucins and interacting proteins from other proteins. Void fractions (fraction 7–11) were pooled and subjected to proteomic analysis. Samples were reduced with 10 mM DTT for 2h at 37°C and alkylated with iodoacetemide at a final concentration of 25 mM and subjected to a HiTrap desalting column (G25- 2x5; GE Healthcare, Cleveland, USA) to exchange buffer with a 50 mM ammonium bicarbonate (pH 8.0) digestion buffer. Samples were then digested with trypsin at 37°C overnight. The digests were dried down with a vacuum evaporator to remove bicarbonate salts. The digest peptides were resolubilized in 20 μzL of 0.1% formic acid water or were stored at -30°C until LC-MS/MS experiments.

### Mass spectrometry

Peptides were separated by ultrahigh pressure liquid chromatography using a Dionex Ultimate 3000 RSLC Nano system coupled to a hybid quadrupole orbitrap mass spectrometer with a Nano spray source (Q-Exactive, Thermo Fisher, Bremen, Germany). For liquid chromatography, one microliter of the sample was loaded into a trap column Acclaim PepMap 2 cm × 75 μm i.d., C18, 3 μm, 100 A (Dionex) at 5 μl/min with aqueous solution containing 0.05% (v/v) trifluoroacetic acid and 2% acetonitrile. After 7 minutes, the trap column was set on line with an analytical column Acclaim PepMap RSLC 15 cm × 75 μm i.d., C18, 2 μm, 100 A (Dionex) with a linear gradient of 4–30% solvent B (99.9% acetonitrile with 0.1% formic acid) over 157 min with a constant flow of 300 nL/min. Eluted peptides were analyzed by a data-dependent top 10 method dynamically choosing the most abundant precursor ions from the survey scan (300–1650 Th) for HCD fragmentation. For MS scan, data were acquired at a resolution of 70,000 at m/z 200, target AGC value of 1e6, and maximum fill times of 80 ms. For the MS/MS scan, data were acquired at a resolution of 17,500 at m/z 200, target AGC value of 1e5, and maximum fill times of 80 ms. Dynamic exclusion was set to 20 seconds. The processed data were searched against the National Center for Biotechnology Information (NCBI) non-redundant protein database (*Canis lupus*, version 10242013) using the Proteome Discoverer (Thermo Scientific) search engine. Parameters used for the Protein Discoverer search were as follows: taxonomy *Canis lupus*, 0.2-Da mass accuracy for parent ions and 0.3-Da accuracy for fragment ions, allowance for one missed cleavage, and use of carbamidomethyl-cysteine and methionine oxidation as fixed and variable modifications, respectively.

### Data analysis

Proteins identified from the mucus samples were quantified using a label-free method termed the normalized spectral index (SIN). SIN is defined as the cumulative fragment ion intensities for all spectra counted for a protein (SI) normalized by the sum of SI over all proteins and by the length of the protein. Label-free quantitative analysis of mucin and interacting proteins was performed using the MaxQuant 1.3.0.5 for four biological replicates from each group. The intensities of identified peptide ions for each protein were summarized as protein intensities. The protein intensities were normalized to total intensity of all identified proteins in each run before comparison. Statistical significance levels between the normal mucus group and the mucocele group were determined by student's t-test (2-tailed, type 2). Proteins with a *p*-value smaller than 0.05 were considered to be significantly increased or decreased. For quantification, the average protein intensities for four biological replicates were compared to determine protein ratios.

## Results

### Mucocele formation is distinguished by accretion of gelatinous content that eventually obstructs or ruptures the gallbladder

Over a 28 month period, fresh samples of gallbladder mucocele mucosa and content were obtained intra-operatively from 11 client owned dogs immediately following surgical cholecystectomy and 1 dog immediately after owner-requested euthanasia. The dogs ranged in age from 2 to 14 years (median, 9 years). Breeds of dog represented included Shetland sheepdog (n = 3), Cocker spaniel (n = 2), Cairn terrier, Chihuahua, German shepherd dog, Italian greyhound, pug, Shih Tzu, and Scottish terrier (n = 1 each). For comparative purposes, gallbladder mucosa and content were obtained immediately following death of 8 clinically healthy research dogs undergoing euthanasia for reasons unrelated to this study. The dogs ranged in age from 2 to 8 years (median, 5 ½ years). Breeds of dog included Beagle (n = 4), mongrel (n = 3), and Foxhound (n = 1).

**Fig 1:**
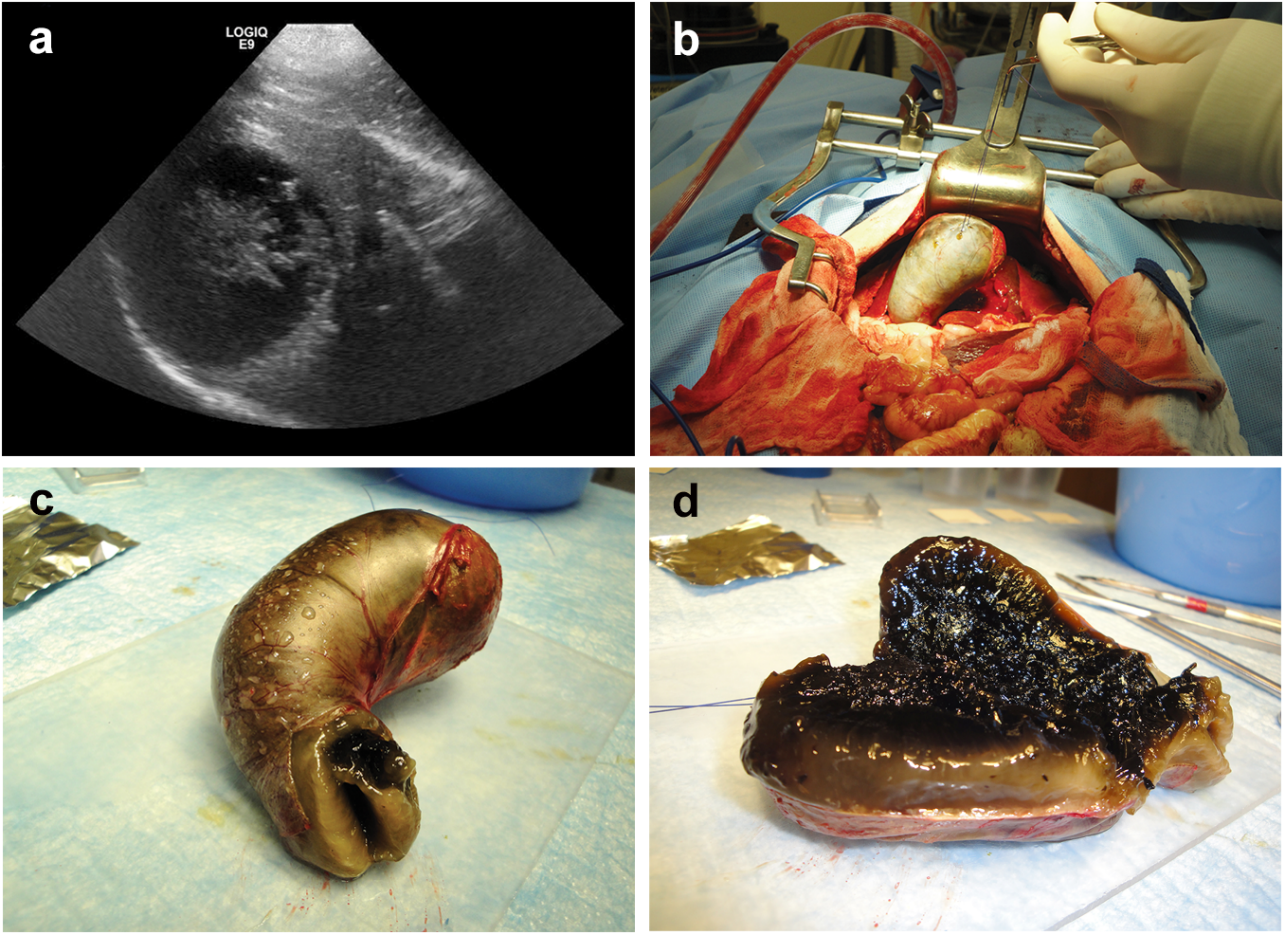
Ultrasonographic, intra-operative and post-surgical appearance of a gallbladder mucocele in an 11 year old male dog. (**a**) Characteristic ultrasonographic appearance of a gallbladder mucocele consisting of an enlarged gallbladder containing immobile bile containing a stellate pattern. (**b**) Midline laparotomy demonstrating surgical isolation prior to removal of a gallbladder mucocele from a clinical canine patient. (**c, d**) Intact, excised gallbladder mucocele demonstrating turgid distension of the gallbladder wall and content characterized by highly gelatinous viscoelastic mucus.

**Fig 2:**
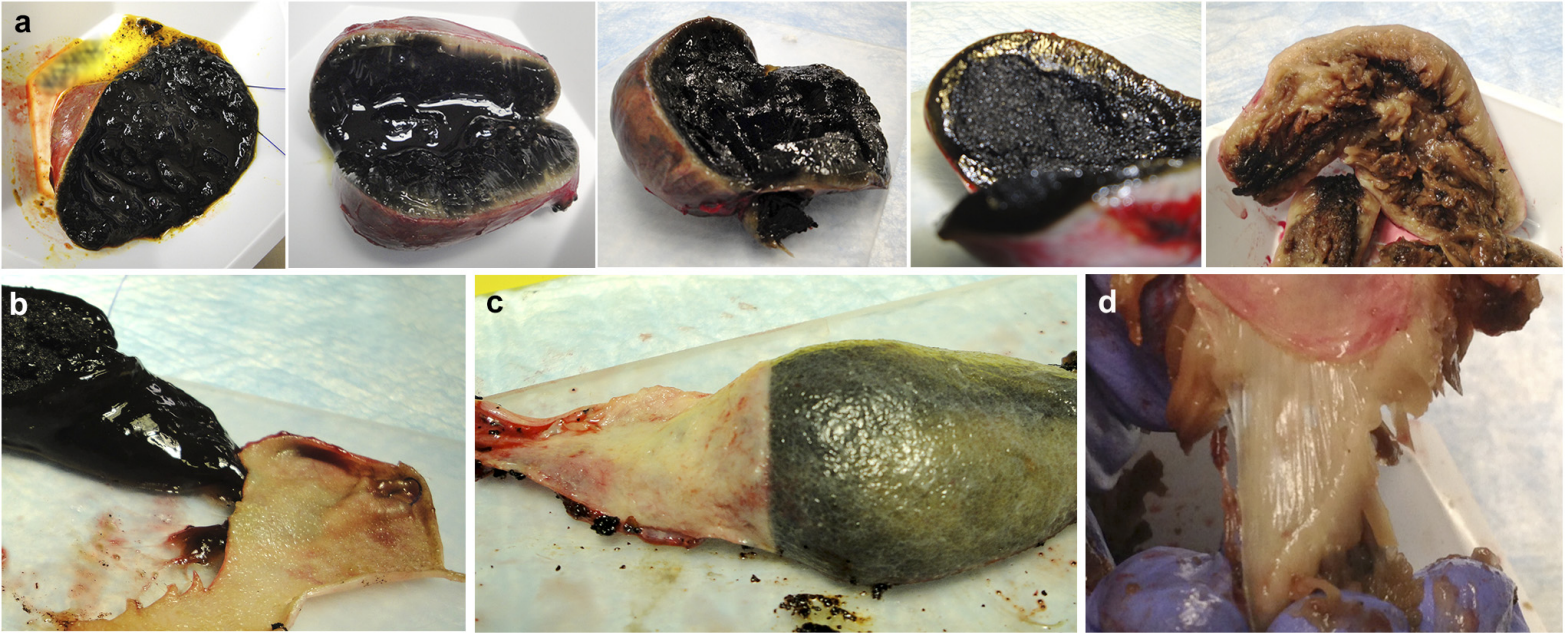
Variation in gross appearance of gallbladder content in dogs with mucocele formation. (**a**) Gross appearance of the content of the gallbladder from 5 different dogs having a gallbladder mucocele. With decreasing hydration of the gallbladder content, increasing association of the mucus with the gallbladder mucosa was observed and ranged from loosely adherent (**b**), to peel-able (**c**), to firmly attached in a manner reminiscent of a ligament (**d**).

Commonly abnormal clinical pathological features of dogs diagnosed with a gallbladder mucocele that were included in the study are shown in **Table 1**. In each case a diagnosis of gallbladder mucocele was presumed on the basis of characteristic trans-abdominal ultrasonographic appearance of the gallbladder and confirmed by gross and light microscopic examination of the gallbladder and contents (**Fig 1**). Additional light microscopic abnormalities within the gallbladder mucosa were documented in 8 dogs and consisted of thrombosis or necrosis attributed to gallbladder infarction (4 dogs), minimal to mild lymphoplasmacytic infiltrates (3 dogs), and a few very small clusters of neutrophils (1 dog). Results of aerobic and anaerobic bacterial culture of gallbladder content was available for 9 (75%) dogs diagnosed with a gallbladder mucocele. Two of these dogs had positive culture results reported as *Streptococcus gallolyticus* and *Staphylococcus hemolyticus* (thioglycolate broth only), respectively. Inflammatory infiltrates were not documented in the gallbladder mucosa of either culture-positive dog. For the 3 dogs in which culture results were not reported, cultures were either cancelled (2 dogs) or not requested by the attending clinician (1 dog) because the dog had died or was euthanized. Cultures of gallbladder content were not performed on any control dogs. Gross contents of the gallbladder of dogs with mucocele formation varied from moist and soft to increasingly dehydrated and rubbery. The physical association between the gallbladder content and mucosal epithelium ranged from loose and easily separable to firmly adhered in a manner similar in appearance to that of a viscoelastic ligamentous attachment (**Fig 2**).

**Table 1:** Results of selected clinical pathological variables assessed in 12 dogs that were diagnosed with a gallbladder mucocele and included in this study.

Clinical Pathological Variable	Median	Range	Number (%) of dogs with abnormal value	Reference range
**Complete blood cell count**				
Packed cell volume (%)	39	26–51	6/12 (50)	39–58
Plasma protein (g/dl)	7.1	4.3–10	7/12 (58)	6.1–7.5
Polymorphonuclear leukocytes (× 10^3^/μl)	12.605	4.181–30.628	7/12 (58)	2.841–9.112
Bands (× 10^3^/μl)	161	0–5.281	8/12 (67)	0.0–0.0
Immature granulocytes (× 10^3^/μl)	0	0–270	1/12 (8)	0.0–0.0
Platelets (× 10^3^/μl)	315	70–937	5/12 (42)	191–468
**Serum biochemical analysis**				
Alkaline phosphatase (IU/L)	968	24–5575	10/12 (83)	16–140
Alanine transaminase (IU/L)	491	20–1403	11/12 (92)	12–54
Gamma-glutamyl transferase (IU/L)	29	0–282	10/12 (83)	0–6
Total bilirubin (mg/dl)	0.6	0–11.2	7/12 (58)	0–0.2
Cholesterol (mg/dl)	366	148–875	7/12 (58)	124–344
Lipase (IU/L)	103	16–2332	4/12 (33)	12–147
Amylase (IU/L)	700	281–4817	2/12 (17)	236–1337

### Gallbladder epithelium acquires a mucin-secretory phenotype during mucocele formation

**Fig 3:**
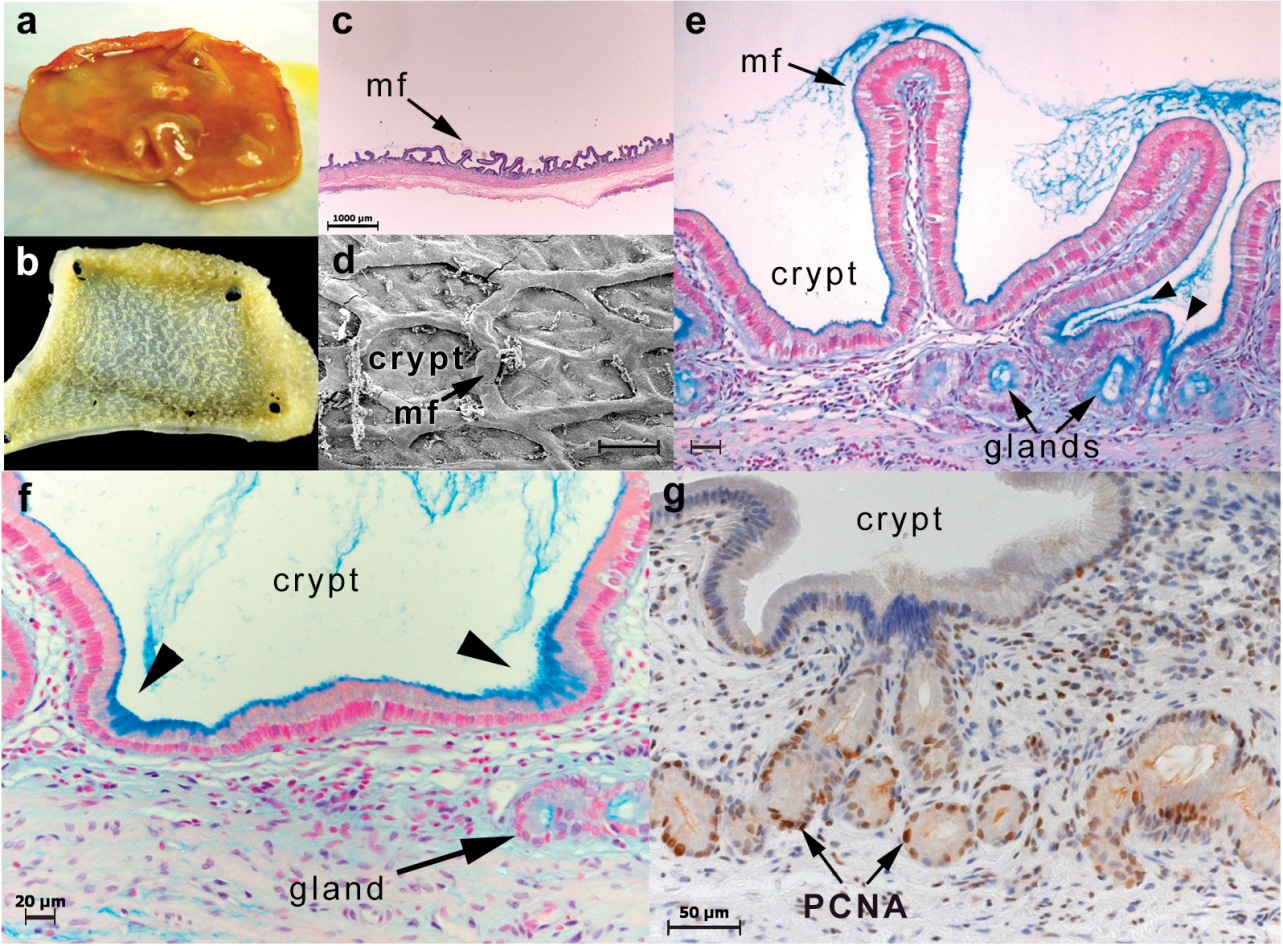
Architecture of normal canine gallbladder mucosa. (**a**) Gross appearance of the gallbladder mucosa reveals a small quantity of loosely viscous mucoid content. This mucus is produced by mucosa comprised of a reticular framework of mucosal folds (**b**, gross photography; **c**, hematoxylin & eosin [bar = 1,000 μm]; **d**, scanning electron microscopy [bar = 250 μm]). (**e**, **f**) Gland openings (arrowheads) lead to sub-epithelial invaginations (glands) in which there is an enrichment of mucin-secreting epithelial cells (alcian blue stain pH 2.5; bar = 20–50 μm). (**g**) Immunostaining of the gallbladder mucosa for the presence of proliferating cell nuclear antigen (PCNA) identifies antigen-positive epithelial cells residing predominantly in sub-epithelial glands (bar = 50 μm). mf–mucosal fold; glands–sub-epithelial glands; PCNA–proliferating cell nuclear antigen. Figure representative of 2 to 8 dogs per imaging modality.

**Fig 4:**
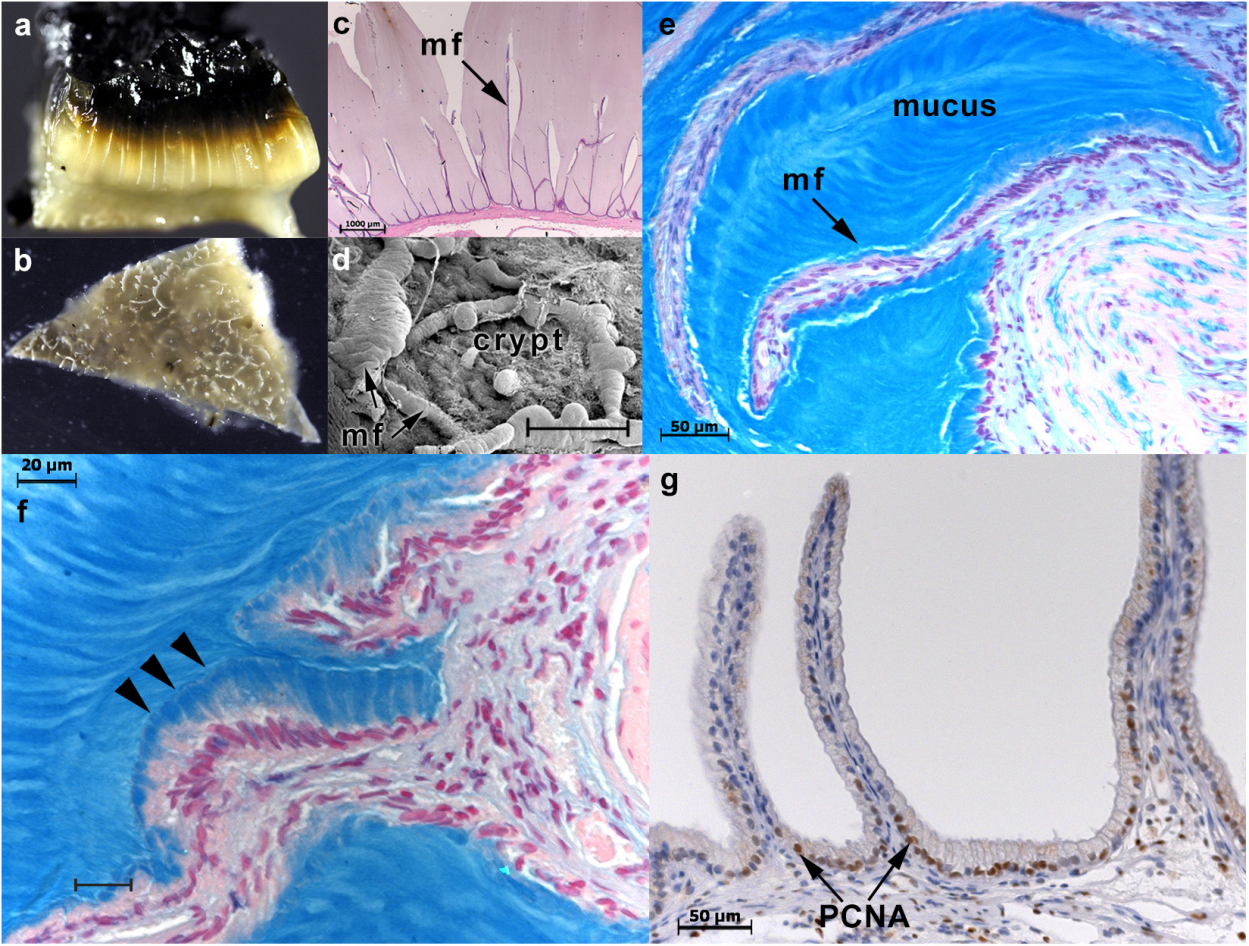
Changes in architecture of the gallbladder mucosa in dogs with mucocele formation. (**a**) Gross appearance of the gallbladder mucosa in mucocele formation reveals copious accumulation of gelatinous mucoid content. The mucoid content transitions from pale at the base and along the length of the mucosal folds (mf) to black near the lumen (presumably due to coloration imparted by lumen bile constituents). After removal of mucus, gross photography (**b**), brightfield microscopy (**c**; hematoxylin and eosin [bar = 1,000 μm]) and scanning electron microscopy (**d**; bar = 200 μm) of the lumen mucosa reveals a reticular framework of mucosal folds that are more flattened, elongate and “frond-like” compared to normal gallbladder. Sub-epithelial glands are not observed (**e**, alcian blue pH 2.5 [bar = 50 μm]). However all epithelial cells appear to be producing mucin (**f**, arrow heads and linear striations of mucin extend from each individual epithelial cell into the lumen content (alcian blue pH 2.5; bar = 20 μm)). (**g**) Immunostaining of the gallbladder epithelium identifies PCNA positive cells that reside along the surface and base of mucosal folds (bar = 50 μm). mf–mucosal fold; PCNA–proliferating cell nuclear antigen. Figure representative of 2 to 12 dogs per imaging modality.

In dogs as in humans, mucin-secreting gallbladder epithelial cells variably reside in gland-like structures that consist of short tubular invaginations of epithelium into the lamina propria[20]. It is frequently presumed that mucocele formation in dogs is associated with hyperplasia of these glands resulting in variable descriptions of the condition as involving a mucinous or cystic hyperplasia[9–12,14]. To determine the role of the glandular epithelium in the pathogenesis of mucocele formation, we examined the location of mucin-secretory and proliferating cell phenotypes in normal and mucocele gallbladder mucosa. Normal gallbladders were characterized by mucosal folds that anastomose to form polygonal structures lined by a columnar epithelium (**Fig 3**). Sub-epithelial glands were commonly identified in normal mucosa and both mucin-secretory and proliferating cell nuclear antigen (PCNA)-positive epithelial cells were concentrated in these regions. In contrast, in the mucocele gallbladder the mucosal folds appear flattened and consist of slender fronds of epithelium extending into the congealed mass of mucus content (**Fig 4**). Sub-epithelial glands were not observed in mucocele gallbladder mucosa and were replaced by a surface columnar epithelium containing fewer numbers of PCNA positive cells that extended only a short distance up from the base of the mucosal folds. Large amounts of mucin could be observed in the apical cytoplasm of all gallbladder epithelial cells and in some gallbladders mucin secretion could be visualized as stacked accretions of mucin extending from each individual cell into the gallbladder lumen.

### Lumen content of the gallbladder mucocele is histochemically characterized by the presence of highly glycosylated acidic mucins

**Fig 5:**
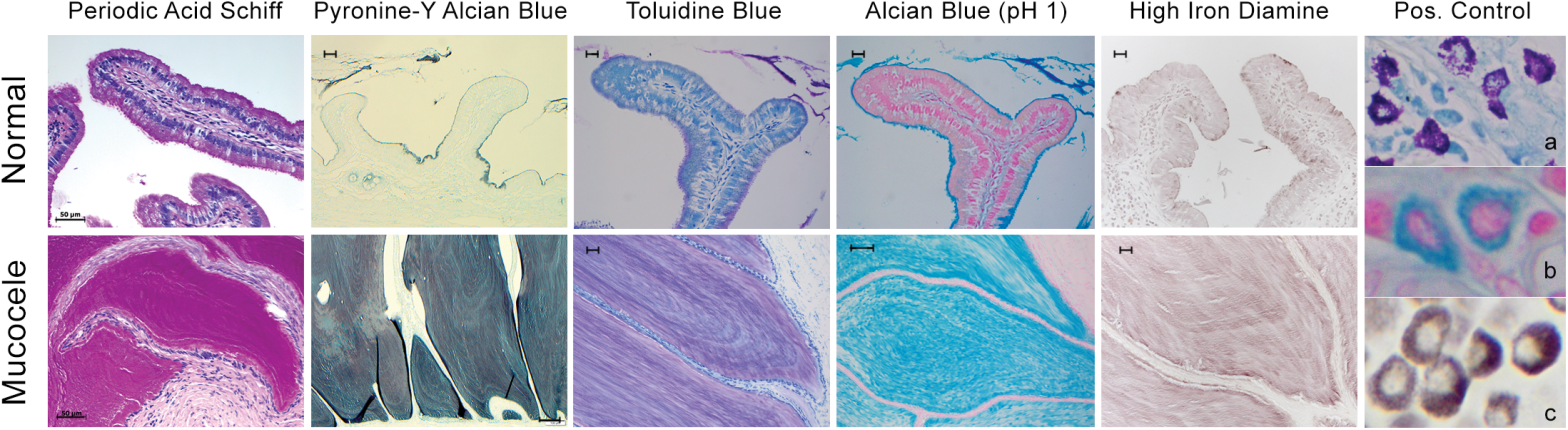
Histochemical characterization of mucin oligosaccharides identifies an admixture of carboxylated and sulfated carbohydrates during gallbladder mucocele formation. Intense staining with periodic acid Schiff (bar = 50 μm) and alcian blue (pH 2.5; Fig 4E and 4F) identify the presence of highly glycosylated mucin comprised of anionic polysaccharide chains. Heterogenous reactivity to the sulfate-group selective stains pyronine-Y (bar = 20 μm top panel and 100 μm bottom panel), toluidine blue, alcian blue at a pH of 1, and high iron diamine (bar = 20 μm) indicate presence of an admixture of sialated and sulfated mucin. Positive controls for intense sulfated glycoprotein staining include canine mast cells (containing heparin sulfate granules) stained with toluidine blue (panel a), alcian blue (pH = 1.0; panel b) and high iron diamine (panel c). Figure representative of 2 to 4 dogs per imaging modality.

Mucins are composed of a central apomucin protein core to which abundant side chains of glycan are covalently linked by means of O-glycosylation to serine and threonine amino acid residues. Sialic acid and sulfate groups are often added terminally to these glycans. Due to the influence of glycan content and terminal additions on the ionic composition, charge, and hydration of mucin, we characterized the mucin carbohydrates present in normal and mucocele gallbladder mucin on the basis of histochemical reactivity (**Fig 5**). Based on intense staining with periodic acid Schiff and alcian blue (pH 2.5) the presence of highly glycosylated mucin was confirmed and identified as composed of anionic polysaccharide chains. Based on a heterogenous histochemical reactivity to the sulfate-group selective stains toluidine blue, high iron diamine, pyronine-Y, and alcian blue at a pH of 1, the presence of alternating layers of predominantly sialated versus sulfated mucin in the lumen mucus was supported. Hyaluronic acid was not detected in either normal or gallbladder mucocele content based on failure of bacterial or porcine hyaluronidase to diminish staining of the mucin with alcian blue (data not shown).

### Mucocele formation is associated with excess secretion of the gel-forming mucins Muc5ac and Muc5b

Six different apomucins are reported to be differentially expressed by normal human and mouse gallbladder epithelial cells including two membrane-bound mucins (MUC3 and MUC1), and four secreted gel-forming mucins (MUC2, MUC5AC, MUC5B, and MUC6)[21,22]. The identity of mucins secreted by canine gallbladder epithelium has not been previously reported. To determine the role of mucins in mucocele formation, we identified the mucins present and ascertained their quantity and properties within the mucus content of normal and mucocele gallbladders obtained from dogs. Using gel-filtration chromatography to separate the mucins from non-interacting proteins followed by mass spectrometry, the gel forming mucin Muc5b and less so Muc5ac was identified as the major mucin secreted by normal canine gallbladder epithelium (**Table 2**). Mucins isolated from the gallbladder mucus of dogs with mucocele formation demonstrated a 17-fold increase in content of Muc5ac (p = 0.012) and a non-significant 2.5 fold increase in content of Muc5b (**Table 2**). This represents a 7-fold increase (p = 0.048) in the ratio of Muc5ac to Muc5b in the gallbladder mucus content of dogs with mucocele formation.

**Table 2:** Differential expression of proteins associated with the large polymeric mucin-rich fraction of mucus isolated from normal compared to mucocele gallbladders as determined by mass spectrometry.

Protein Description (access gi#[Canis lupus familiaris] and name)	Average Total Intensity		Fold Increase	t test p-value
	Normal (n = 4)	Mucocele (n = 4)		
**Mucins and related proteins**				
gi|359321890 Mucin-5ac	6.883E+8	1.148E+10	16.6	0.012
gi|345783652 Mucin-5b	2.905E+10	7.015E+10	2.5	0.163
Ratio mucin-5ac/5b	2.37E-2	16.36E-2	6.9	0.048
gi|359318821 IgGFc-binding protein	1.383E+10	3.251E+11	23.5	>0.0001
gi|345792551 Deleted in malignant brain tumors 1 protein	6.912E+7	1.576E+9	22.8	>0.0001
gi|156875884 Trefoil factor family peptide 3	2.070E+8	3.438E+9	16.6	0.003
gi|345802838 Polymeric immunoglobulin receptor	1.923E+9	2.232E+10	11.6	0.0028
gi|73953129 Pulmonary surfactant-associated protein D isoform 1	Nd	6.103E+8		0.036
gi|73964953 Galectin-3-binding protein	Nd	2.019E+7		0.036
**Immunoglobulins (Ig)**				
gi|345779666 Ig J chain isoform 1	3.982E+8	1.093E+10	27.4	>0.0001
gi|124390009 IgM heavy chain constant region CH2	7.521E+7	1.729E+9	23.0	0.015
gi|124390007 IgM heavy chain constant region CH1	2.653E+7	6.094E+8	23.0	0.015
gi|164452882 Ig kappa light chain variable region	2.980E+7	5.174E+8	17.4	0.015
gi|208342271 Ig heavy chain variable region, partial	9.906E+6	1.460E+8	14.7	0.04
gi|208342106 Ig heavy chain variable region, partial	1.428E+7	8.151E+7	5.7	0.052
gi|1096664 IgA heavy chain constant region	6.781E+9	2.887E+10	4.3	0.0019
**Complement components/factors**				
gi|359322249 Complement C3	2.426E+8	2.302E+9	9.5	0.002
gi|359320893 Complement C4-A	2.753E+7	2.371E+8	8.6	0.02
gi|73997271| Complement C1r subcomponent isoform1	Nd	8.884E+7		0.039
gi|345803075 C4b-binding protein alpha chain	Nd	4.308E+7		0.109
gi|74002140 Complement factor I isoform 3	Nd	3.242E+7		0.11
gi|57091989 Complement component C8 gamma chain	Nd	2.550E+6		0.122
gi|73997275 Complement C1s subcomponent isoform 2	Nd	3.368E+7		0.134
gi|74005944 Complement factor H isoform 2	Nd	2.550E+6		0.15
**Proteases/antiproteases**				
gi|119637732 Alpha-1 antitrypsin	7.713E+6	2.820E+8	36.5	0.0017
gi|73946216 Plasminogen	6.065E+6	1.419E+8	23.4	0.005
gi|73964432 Alpha-1-antichymotrypsin	5.411E+6	9.483E+7	17.5	0.0087
gi|73967363 Alpha-2-antiplasmin isoform 2	Nd	2.309E+7		0.029
**Other**				
gi|73988725 Hemopexin	3.410E+7	1.110E+9	32.5	0.005
gi|22531688 Serum albumin	1.544E+9	1.242E+10	8.0	0.01
gi|345781768 Fibrinogen-like 1	6.725E+6	3.330E+7	5.0	0.008

### Gallbladder mucocele mucus is characterized by abnormal quantity, macromolecular properties, and sedimentation behavior of Muc5b

**Fig 6:**
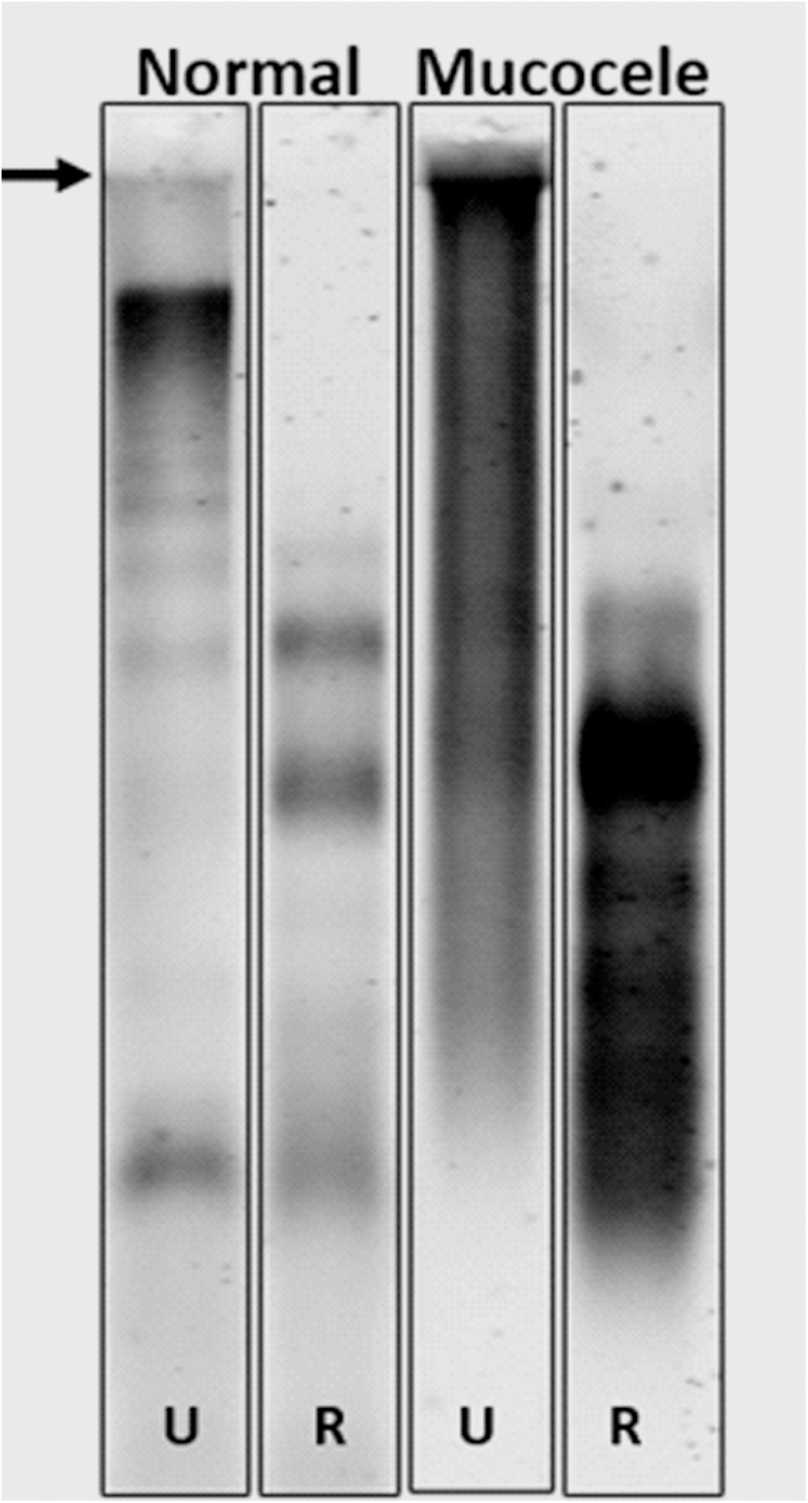
A typical Muc5b immunoblot of normal and mucocele mucus. An aliquot from each mucus sample was solubilized in 6M urea and subjected to agarose (0.7%) gel electrophoresis under unreduced (U) and reduced (R) conditions. The gel illustrates the difference in quantity and macromolecular properties of the mucin between the samples. A significant amount of Muc5b reactivity observed in the origin (arrow) in unreduced mucocele mucus suggests the presence of a very large molecular weight population of the mucin. After reduction, mucocele mucus is liberated into multiple Muc5b glycoforms whose mobility variance can be attributed to differences in glycosylation (e.g. sialyation, sulfation)[1]. Figure is representative of at least 3 independent experiments.

Samples of mucus from normal and mucocele gallbladders were examined for the presence and macromolecular organization of Muc5b mucin by solubilizing the mucus samples and subjecting the proteins to agarose gel electrophoresis, under unreduced and reduced conditions, followed by immunoblotting (**Fig 6**). In normal gallbladder mucus, unreduced Muc5b demonstrated a typical band of immunoreactivity just beneath the origin followed by a multimeric migration pattern. After reduction, Muc5b from normal mucus was liberated into subunits with two apparent glycoforms. In mucocele gallbladder samples, unreduced Muc5b demonstrated retention of a large portion of immunoreactivity at the origin with smearing throughout the remainder of the lane, suggesting the presence of a very large multimeric Muc5b structure. After reduction, Muc5b from the abnormal mucus showed a different migration suggesting substantial changes in the glycosylation pattern of the subunits of the mucin.

**Fig 7:**
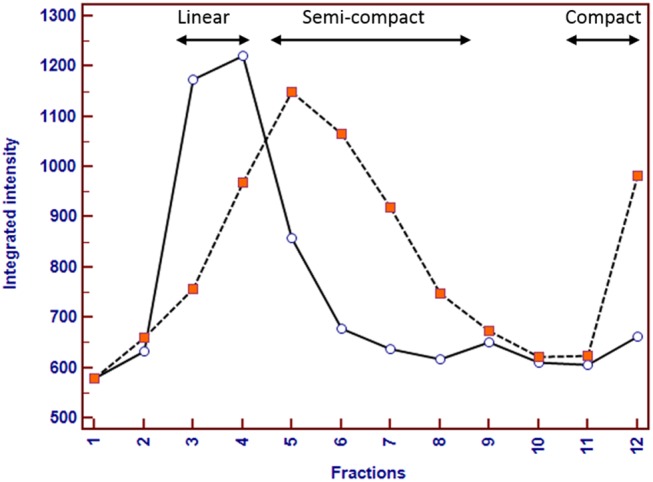
Distribution of Muc5b forms in normal (solid line) and mucocele (dashed line) mucus. Samples were solubilized in 4M GuHCl and layered onto a linear GuHCl gradient (6-8M) and subjected to rate-zonal centrifugation. Fractions were unloaded from the top of the centrifugation tube and subjected to gel electrophoresis and Muc5b immunoblotting. A representative separation and size distribution of Muc5b indicates that in normal mucus most of the Muc5b is found in the low density region as linear form (fractions 3–5). In mucocele mucus however, Muc5b is observed mainly in rapidly sedimenting compact granular form (fraction 12) and semi-compact form (fractions 5–8) suggesting a defective unpacking process (maturation to the linear form). Figure is representative of at least 3 independent experiments.

To further characterize the macromolecular organization of Muc5b in mucus from normal gallbladders compared to those with mucocele formation, mucus samples were solubilized and layered onto a linear guanidium HCl gradient (6-8M), and fractionated by means of rate-zonal ultracentrifugation. Typically, MUC5B in human saliva distributes into three different regions representing linear, semi-compact, and compact molecular conformations based on mass, size, and shape[2]. Here, for normal gallbladder mucus, most of the Muc5b was found in the low density region as the linear form (fractions 3–5). In contrast, for mucocele gallbladder mucus, Muc5b was found mainly in the rapidly sedimenting compact granular form (fraction 12) and semi-compact form (fractions 5–8) (**Fig 7**).

### Exocytosis of mucin by gallbladder mucocele epithelium is associated with abnormal unpacking of mucin from granule matrix

**Fig 8:**
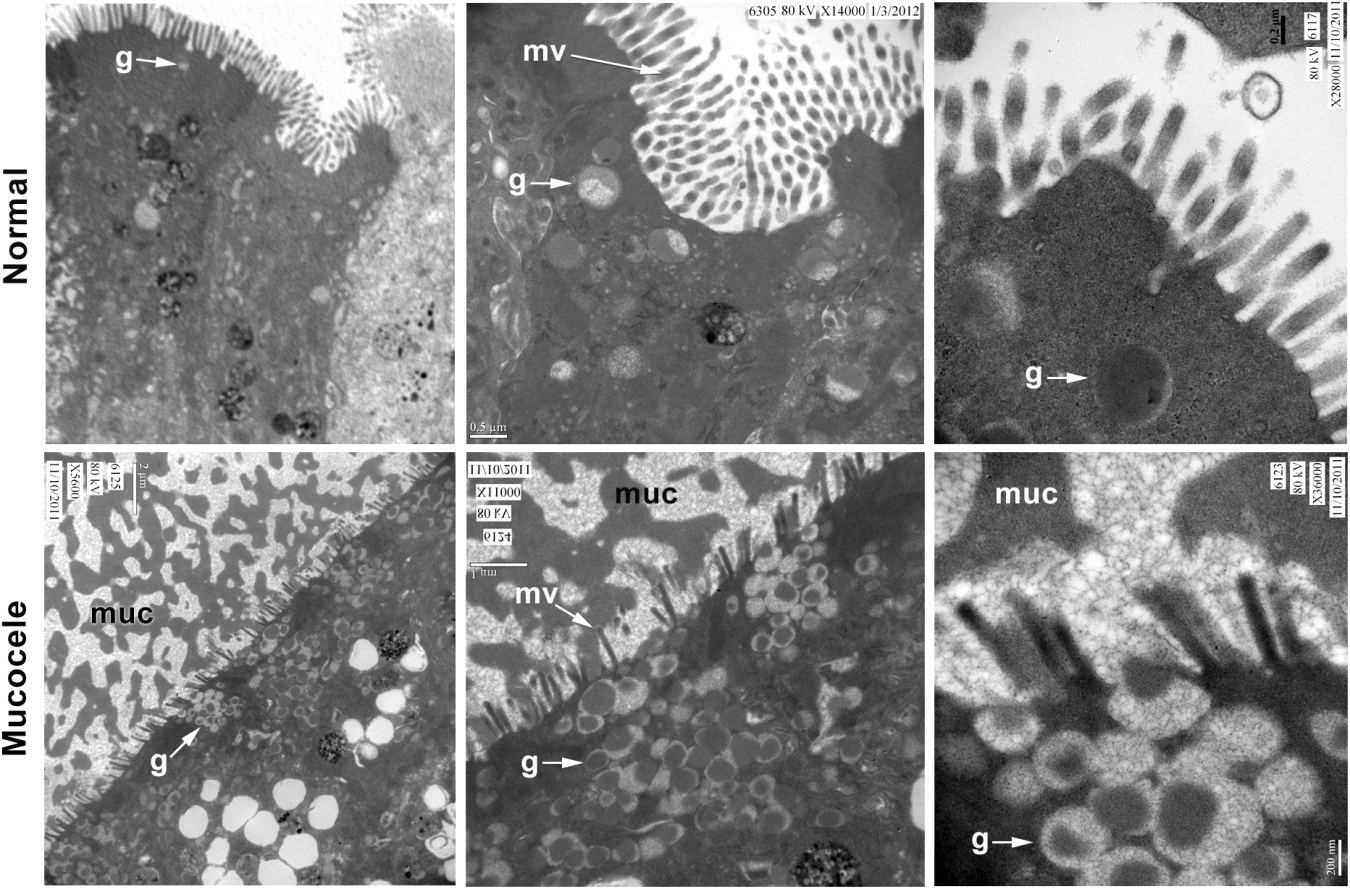
Transmission electron microscopy of gallbladder epithelial cells from normal and mucocele gallbladder mucosa. Compared to normal gallbladder epithelial cells, mucocele epithelial cells contain copious numbers of mucin granules (g) and failure to un-package granule content following exocytosis. g–mucin granule; muc–mucus; mv–microvilli. Left panels bar = 2 μm; middle panels bar = 0.5–1 μm; right panels bar = 200 nm. Figure representative of 3 mucocele and 2 control dog gallbladders.

Mucins are synthesized in the endoplasmic reticulum and Golgi and stored in secretory granules within the mucous epithelial cells. Upon exocytosis, mucin granules rapidly disperse to form a protective mucus blanket. To gain further insight into the abnormal behavior of the mucin upon secretion by gallbladder epithelium during mucocele formation, we performed an ultrastructural examination of mucin exocytosis (**Fig 8**). Compared to normal dogs, the gallbladder epithelium of dogs with mucocele formation was characterized by copious numbers of mucin granules occupying a majority of the apical cytoplasm. Granule contents in mucocele epithelial cells were highly compact, electron-dense and surrounded by a complex filamentous network. Once in the lumen, granule contents appeared to merge with large conglomerations of earlier granules, none of which demonstrated dissolution. These conglomerations of mucin remained interconnected to each other and with the epithelial microvilli by direct association with the same filamentous network observed in granule contents prior to their release.

### The proteome of gallbladder mucocele mucus is enriched with mucin cross-linking and innate defense proteins

In addition to mucins, hundreds of other proteins with a broad range of functionalities are secreted onto epithelial surfaces[19]. These proteins are either secreted from surface goblet cells or glandular secretory epithelial cells while some infiltrate is derived from the serum. Some of these proteins can be stored and secreted together with or without mucins and interact directly with them. Interacting proteins may instigate profound effects on mucin organization within the granules[19] as well as their viscosity after release[2]. To date very few studies have been performed to determine the identity of these mucin-associated proteins[19,23]. We therefore undertook a proteomics approach to determine the identity of proteins specifically associated with the large polymeric mucin-rich fraction of mucus isolated from normal compared to mucocele gallbladders. Our rationale was that these proteins might play an important role in mediating pathological effects on mucus behavior during mucocele formation. Using CL2B gel filtration chromatography, large molecular weight mucins were separated from other (non-binding) proteins. Mucins and their binding proteins were then eluted in the void volume (Vo). While none of the globular proteins are large enough to elute in the Vo region, inclusion of large protein-protein complexes near the Vo cannot be ruled out. Approximately 24 proteins were significantly increased in mucocele samples (**Table 2**and **S1 File**). Increases in the mucin-associated proteins trefoil factor (TFF) III, IgGFc binding protein (FCGBP), and deleted in malignant brain tumors 1 (DMBT1) were noteworthy due to their documented ability to cross-link or entangle with gel-forming mucins. Pulmonary surfactant-associated protein D isoform 1 (SP-D) and galactin-3 binding protein were increased significantly in mucocele samples while they were not detected in normal samples. It is also noteworthy that components of the secreted immunoglobulins IgA and IgG along with J chain and polymeric immunoglobulin receptor which are required for IgA and IgG secretion were significantly increased in the mucocele samples. Moreover, complement factors C3 and C4-A were significantly increased while multiple other complement components such as C1r, C1s and C8 and factors I and H were only detected in the mucocele samples. Protease inhibitors, like alpha-1 antitrypsin, alpha-2-antiplasmin and alpha-1-antichymotrysin were significantly increased in the mucocele mucus. Mucocele samples also exhibited an increased concentration of serum associated proteins such as plasminogen, hemopexin, fibrinogen-like 1, and serum albumin.

## Discussion

The underlying cause of gallbladder mucocele formation in the dog is currently somewhat of a mystery. Several predisposing factors such as breed predisposition[11,15], concurrent endocrinopathies[13], and hyperlipidemia[11,15] suggest both a genetic and hormonal/metabolic contribution to disease pathogenesis. However, the breeds of dog affected and endocrinopathies commonly associated with gallbladder mucocele formation have existed long before emergence of the disease as a clinical entity and are not found in all dogs diagnosed with the disease. Accordingly, these are unlikely to be a direct cause, but rather an exacerbating factor to disease pathogenesis. Efforts to link a genetic defect in ABCB4, a hepatocyte canicular membrane phosphatidylcholine flippase, in the Shetland sheepdog was initially promising but later disproven[24]. A theory that poor gallbladder motility[16] causes gallbladder mucocele formation is difficult to prove once the gallbladder is filled with mucus and gallbladder paresis does not result in mucocele formation in people. The disease is not a consequence of common bile duct obstruction[25], however mucus can eventually extend into and obstruct hepatic bile drainage. Increased mucin secretion can be caused by bacterial cholecystitis, however infection of the gallbladder is an inconsistent finding in dogs with gallbladder mucocele formation[9–12,14]. Despite many descriptions of proliferative changes in the gallbladder epithelium[26], there exist no diseases of the gallbladder in people that closely mirror the histological appearance of gallbladder mucocele formation in dogs. The only descriptions of gallbladder mucosa that are similar to gallbladder mucocele formation are in animals experimentally treated with progestins[27–29] or lacking functional cystic fibrosis transmembrane regulatory (CFTR) protein expression[30,31]. Alas, the specific mechanisms underpinning the initiating event of the disease pathogenesis in dogs has remained elusive.

These studies have utilized immunohistochemical, ultrastructural, biochemical and biophysical, and proteomics analysis to provide key mechanistic insights into the pathogenesis of abnormal mucus formation and the syndrome of gallbladder mucocele disease in dogs. First among these is our observation that the gallbladder epithelium acquires a well differentiated mucin-secretory phenotype that collectively contributes to mucocele formation. This finding contradicts the frequent assumption that mucocele formation is a consequence of “glandular hyperplasia” of the gallbladder mucosa. As documented in these studies, absence of sub-epithelial glands and lack of evidence for an overt increase in number of PCNA-positive epithelial cells suggests that glandular epithelial proliferation is not a perpetuating mechanism for excessive mucin secretion. It is worth noting that an early histological description of a similar condition in dogs, commonly presumed to be an ancestral phenotype of the present-day gallbladder mucocele, was identified by the authors as cystic mucinous hypertrophy[32] and not hyperplasia as is commonly quoted[9–12,14]. Accordingly, the elongated fronds of epithelium that are characteristic of a gallbladder mucocele may be surmised to reflect a change in architecture of the mucosal folds and their flattening by insidious accretion of mucus in the lumen content and not a consequence of primary epithelial hyperplasia.

The gallbladder epithelium is protected from the actions of excretory bile by a blanket of secreted mucus. Protective mucus is typically composed of gel forming mucins and additional functional and structural proteins. Proteome studies have identified the presence of 9 different mucins as well as several thousand other proteins in gallbladder bile[33–35]. However, little is known regarding the protein composition of gallbladder mucus. In these studies we isolated the large multimeric (glyco) proteins from mucus obtained from normal and mucocele gallbladders and performed a quantitative proteomic analysis using mass spectrometry. Two gel forming mucins, Muc5b and Muc5ac, were identified as the major constituents of mucus from both normal and mucocele gallbladders. In normal mucus, Muc5b was the major mucin present. In mucocele mucus however, we identified a significant (17 fold) increase in content of Muc5ac mucin. Surprisingly, not a single peptide from Muc2 mucin was identified in either normal or mucocele gallbladder mucus, despite Muc2 being a major gastrointestinal mucin. This finding may suggest that Muc2 is not secreted by normal canine gallbladder epithelia.

The gel-forming mucins are the largest glycoproteins in the body. Their large size, high carbohydrate content, and polymeric structure are vital to formation of a viscoelastic mucus layer that is essential for hydration and protection of mucosal epithelium. Proper protection of each mucosal epithelium depends on differences in the quantity and properties of the gel-forming mucins present. In the lung, the ratio of MUC5AC to MUC5B is critical in establishing a fine balance between protection and pathogenesis. In normal lung secretions, this ratio is 0.04 while in disease conditions such as chronic bronchitis it can increase 10-fold to 0.4 (unpublished data, MK). In this study, the ratio of Muc5ac to Muc5b was 0.023 in normal gallbladder mucus and increased significantly to 0.163 in mucus from gallbladder mucoceles. Although speculative, an increase in Muc5ac expression and consequently the ratio of Muc5ac to Muc5b is likely to contribute to gallbladder mucocele formation by promoting a more cross-linked, viscous and rubber-like mucus.

These studies also identified proteins in mucocele mucus that are noteworthy for their ability to cross-link or entangle mucin chains. The gel-forming mucins are assembled into large disulfide-linked polymers by means of cysteine-rich von Willebrand factor-like amino acid domains. Formation of disulfide cross-links with other proteins is a major factor in influencing mucus gel behavior. Two mucin cross-linkers, FCGBP and TFFIII[36] were increased 24 and 17-fold respectively in gallbladder mucocele mucus. Another mucin-interacting protein DMBT1[37] was increased 23-fold. FCGBP, TFFIII, and DMBT1 are secreted by intestinal epithelial and goblet cells[38], and mucin producing biliary[6,38,39] and respiratory epithelial cells[37,38,40] where their interaction with mucin increases the viscosity and structural integrity of mucus[41,42]. Significant increases in Muc5ac mucin in the presence of these proteins is likely to promote abnormal biophysical properties of the mucus observed in gallbladder mucoceles. The influence of trefoil factor proteins on mucin viscosity is particularly dramatic. In sufficient quantities, TFFII can transform a liquid solution of gastric mucin into a highly viscous, semi-solid gel[41,42]. Based on their known functions, TFFIII, FCGBP, and DMBT1 are likely to interact cooperatively with mucins to promote epithelial defense[43,44]. Trefoil factor proteins possess a broad range of activity including anti-apoptotic effects, promoting epithelial restitution, and immune modulation. FCGBP is a unique mucin-like protein that was described as binding to the Fc portion of IgG and inhibits IgG-mediated complement fixation[38], while DMBT1 functions as a broadly specific pattern recognition protein that directly interacts with IgA and complement and is capable of agglutinating microbial pathogens[45]. DMBT1 directly interacts with pulmonary surfactant-associated Protein D (SP-D)[46] which was observed only in mucus from gallbladder mucoceles. SP-D functions as a clearance mechanism for apoptotic cells and DNA and as an agglutinin/opsonin for a variety of microorganisms by virtue of broad-spectrum globular carbohydrate recognition domains[47]. Significant increases in complement components, IgA (4-fold) and IgM (23-fold), polymeric immunoglobulin receptor (PIgR)(12-fold), and immunoglobulin J chain (27-fold) suggest an exuberant co-secretion of immune system effector proteins by gallbladder epithelial cells during mucocele formation. The instigating cause for secretion of these proteins remains unclear. There was no or minimal evidence of inflammatory infiltrate in the gallbladder mucosa of these dogs. Bacterial infection of mucocele gallbladders is an inconsistent finding in dogs, however two of the analyzed mucus samples were positive for growth of *Streptococcus gallolyticus* and *Staphylococcus haemolyticus* (growth in broth only) respectively. The analysis of the proteomic results from mucocele mucus from gallbladders with positive versus negative bacterial growth indicated that although some proteins, such as Muc5ac and FCGBP were slightly more increased in positive samples the increase was not found to be significant. A limitation of this study is that bacterial culture of bile was not completed in some dogs having gallbladder mucocele formation nor were cultures performed in any of the control dogs. Given the innate immune function of many of the mucin-interacting proteins identified in mucocele mucus, future studies designed to examine the gallbladder and intestinal microbiome of dogs with mucocele formation compared to control dogs is justified.

Mucosal surfaces are hydrated continuously by fluid secretions. Increased mucus solids and mucin concentrations increase the osmotic pressure and therefore affect the distribution of water between the mucus and epithelial layer[18]. Defective fluid secretion and/or hyper-absorption will further increase the concentration of the mucus biomolecules. An increase in the osmotic pressure of the mucus will eventually cause adhesion of the mucus layer to the epithelial surface. This will immobilize the mucus layer and likely contributes to the pathogenesis of gallbladder mucocele formation. Ultra-structural examination of mucocele gallbladder mucosa demonstrated abnormal un-packaging, i.e. compromised unfolding, of the secretory mucin granules following exocytosis from the surface epithelial cells. Gel forming mucins are stored in secretory granules in the presence of high calcium and a low pH that keeps them in a highly condensed and compact state. After granule release, mucins rapidly unfold and expand from the compact granular form to a linear form that provides an appropriate matrix gel to the mucosal surface[2]. This so-called mucin maturation process is mediated by an exchange of divalent calcium ions with extracellular monovalent sodium ions which causes water molecules to move into and hydrate the mucus gel. Calcium is also important for multimerization of MUC2 and MUC5B mucins via their non-glycosylated *N*-terminal region, which can be reversed by removing calcium from the multimer[48,49]. The maturation process depends on the environment being protected (e.g. lung, gallbladder, stomach, colon) and is essential for proper function of each individual mucosal system. For instance, the lung requires a transportable mucus and is predominantly composed of mucins in linear form[19] while in stomach the firm mucus layer is composed mostly of compact “not matured” mucins and is required to protect the mucosa from highly acidic gastric fluid. To determine the dominant form of mucin in normal gallbladder mucus and whether this form is altered in mucus from mucocele gallbladders we performed a rate-zonal centrifugation. In normal mucus, the distribution in molecular conformation of Muc5b revealed that most of the mucin was found in the low density region as linear form (fractions 3–5). This is similar to what is found in normal saliva and normal lung secretions[23]. In mucocele samples however, Muc5b was found mainly in the rapidly sedimenting compact granular form (fraction 12) and semi-compact form (fractions 5–8). This finding suggests a defective maturation/unfolding of Muc5b from the compact granular form to a linear form in mucocele mucus. In addition, the high sulfate content of the mucin secreted by mucocele gallbladder epithelium may contribute to delayed un-packaging of mucin granules. This is because sulfate forms stronger bridges with calcium ions than does carboxylate (sialic acid)[50]. The post-secretory environment that mucins are released into is important for the un-packaging process. If the environment is not properly hydrated or the ionic composition is not optimal, e.g. low bicarbonate, this unfolding process can be compromised[51,52]. It was shown that the gallbladder of newborn pigs and juvenile ferrets with cystic fibrosis is characterized by mucus accumulation[31] and cystic mucosal hypertrophy[30] with uncanny resemblance to that observed in gallbladder mucoceles from dogs. This suggests that an underlying defect in electrolyte, acid-base or fluid transport should be strongly considered as a possible cause of gallbladder mucocele formation.

Taken together, here we conclude that mucocele formation in dogs involves excess secretion by the gallbladder epithelium of gel forming mucins with abnormal properties and characterized by a disproportionally significant increase in Muc5ac relative to Muc5b, defective mucin un-packaging, and presence of a unique composition of mucin-interacting proteins that are capable of dramatically altering the physical and functional properties of mucus. These findings are sufficient to explain the thick and rubbery properties of the mucus, however the inciting cause for the mucin secretion remains unknown. Immobilization and adhesion to the mucosal surface may eventually block epithelial fluid transport and further perpetuate mucocele formation. Alternatively or concurrently, an abnormality in the function of gallbladder epithelial transport mechanisms may be an instigating factor in dogs with mucocele formation based on striking similarities between canine gallbladder mucoceles and gallbladders of piglets and ferrets with cystic fibrosis.

## Supporting Information

S1 File Number of peptides and intensities of the mucins and associated proteins as detected by mass spectrometric analysis of mucus from normal and mucocele gallbladders. (XLSX)
